# Patterns and Drivers of Extracellular Enzyme Activity in New Zealand Glacier-Fed Streams

**DOI:** 10.3389/fmicb.2020.591465

**Published:** 2020-11-19

**Authors:** Tyler J. Kohler, Hannes Peter, Stilianos Fodelianakis, Paraskevi Pramateftaki, Michail Styllas, Matteo Tolosano, Vincent de Staercke, Martina Schön, Susheel Bhanu Busi, Paul Wilmes, Alex Washburne, Tom J. Battin

**Affiliations:** ^1^Stream Biofilm and Ecosystem Research Laboratory, School of Architecture, Civil and Environmental Engineering, École Polytechnique Fédérale de Lausanne, Lausanne, Switzerland; ^2^Systems Ecology Research Group, Luxembourg Centre for Systems Biomedicine, University of Luxembourg, Esch-sur-Alzette, Luxembourg; ^3^Department of Microbiology and Immunology, Montana State University, Bozeman, MT, United States; ^4^Selva Analytics, LLC, Bozeman, MT, United States

**Keywords:** nutrient cycling and limitation, alpine biogeochemistry, microbial ecology, extracellular enzyme activities, ecological stoichiometry

## Abstract

Glacier-fed streams (GFSs) exhibit near-freezing temperatures, variable flows, and often high turbidities. Currently, the rapid shrinkage of mountain glaciers is altering the delivery of meltwater, solutes, and particulate matter to GFSs, with unknown consequences for their ecology. Benthic biofilms dominate microbial life in GFSs, and play a major role in their biogeochemical cycling. Mineralization is likely an important process for microbes to meet elemental budgets in these systems due to commonly oligotrophic conditions, and extracellular enzymes retained within the biofilm enable the degradation of organic matter and acquisition of carbon (C), nitrogen (N), and phosphorus (P). The measurement and comparison of these extracellular enzyme activities (EEA) can in turn provide insight into microbial elemental acquisition effort relative to environmental availability. To better understand how benthic biofilm communities meet resource demands, and how this might shift as glaciers vanish under climate change, we investigated biofilm EEA in 20 GFSs varying in glacier influence from New Zealand’s Southern Alps. Using turbidity and distance to the glacier snout normalized for glacier size as proxies for glacier influence, we found that bacterial abundance (BA), chlorophyll *a* (Chl *a*), extracellular polymeric substances (EPS), and total EEA per gram of sediment increased with decreasing glacier influence. Yet, when normalized by BA, EPS decreased with decreasing glacier influence, Chl *a* still increased, and there was no relationship with total EEA. Based on EEA ratios, we found that the majority of GFS microbial communities were N-limited, with a few streams of different underlying bedrock geology exhibiting P-limitation. Cell-specific C-acquiring EEA was positively related to the ratio of Chl *a* to BA, presumably reflecting the utilization of algal exudates. Meanwhile, cell-specific N-acquiring EEA were positively correlated with the concentration of dissolved inorganic nitrogen (DIN), and both N- and P-acquiring EEA increased with greater cell-specific EPS. Overall, our results reveal greater glacier influence to be negatively related to GFS biofilm biomass parameters, and generally associated with greater microbial N demand. These results help to illuminate the ecology of GFS biofilms, along with their biogeochemical response to a shifting habitat template with ongoing climate change.

## Introduction

Glacier-fed streams (GFSs) are characterized by their low water temperatures, high levels of turbidity and suspended sediment loads, and strong diel and seasonal variability in discharge (Ward, 1994; Uehlinger et al., 2010). The ongoing and widespread increase in glacier mass loss resulting from climate change (Zemp et al., 2015) further adds to the dynamic nature of GFSs through changes in meltwater generation and corresponding dissolved and particulate matter fluxes (Milner et al., 2017; Boix-Canadell et al., 2019). As the climate shifts, increases in meltwater runoff are expected for individual glaciers, but seasonal runoff and particulate fluxes will eventually decline when the diminished ice volume can no longer produce more melt (Milner et al., 2017; Huss and Hock, 2018). This transition, commonly referred to as “peak water,” is projected for heavily glaciated areas in the near future, but may have already passed for more lightly glaciated regions (Bliss et al., 2014). Despite the imminent and ongoing nature of this global transition, relatively little is currently known about the ecology of GFSs in comparison to streams from other ecotypes (Milner et al., 2017; Brighenti et al., 2019), and even less is known about how the structure and function of microbial communities will shift as glaciers shrink.

Biofilms are surface-attached and matrix-enclosed microbial communities that dominate microbial life in many ecosystems (Flemming and Wuertz, 2019), including streams (Battin et al., 2016). In GFSs, benthic biofilms can harbor relatively high biodiversity with representatives from all three domains of life exhibiting various metabolic strategies, including heterotrophic, chemoautotrophic, and photoautotrophic components (Wilhelm et al., 2013, 2014; Van Horn et al., 2016). Photoautotrophic activity may be a particularly valuable energy source for heterotrophs in GFSs given the overall energy-poor environment. However, photosynthesis is strongly and negatively affected by high suspended sediment loads and associated turbidity which attenuates solar radiation in the water column (Uehlinger et al., 2010). At the same time, the production of extracellular polymeric substances (EPS), which “‘glues” together cells and substrata to form the biofilm matrix, may help microbes to better withstand high shear stresses and stabilize sediments (Battin et al., 2016; Roncoroni et al., 2019). Furthermore, EPS has the added benefit of retaining moisture during periodic desiccation (Flemming and Wingender, 2010), and immobilizing, concentrating, and storing nutrients and dissolved organic carbon (DOC) (Freeman and Lock, 1995).

In addition to sediments and water, GFSs also deliver DOC (e.g., Hood et al., 2009, 2015; Singer et al., 2012), nitrogen (N) (Hodson et al., 2005; Wadham et al., 2016; Colombo et al., 2019), and phosphorus (P) (Hodson et al., 2004; Föllmi et al., 2009; Hawkings et al., 2016) to downstream ecosystems—all of them typically at low concentrations. However, concentrations of P generally increase with greater glacier influence in comparison to N, probably due to elevated rates of rock comminution beneath the glacier and dust deposition on glacier surfaces (Elser et al., 2020). Therefore, N limitation is predicted to be common for microbial life in GFSs (Ren et al., 2019; Elser et al., 2020), yet few studies have directly assessed nutrient limitation on the microbial life of GFSs (e.g., Robinson et al., 2002; Kohler et al., 2016), representing a considerable barrier in our understanding of the ecology of these habitat types.

Biofilms play a substantial role in fluvial biogeochemical cycling through their uptake, assimilation, and transformation of carbon and nutrients (McKnight et al., 2004; Battin et al., 2016). Given the low water column concentrations of dissolved C, N, and P typical of GFSs, mineralization of organic matter may be necessary to meet carbon and nutrient demands (Kohler et al., 2018). Extracellular enzymes are critical to the decomposition of organic matter by making complex molecules manageable for heterotrophic microbes to incorporate, therefore playing an important role in biogeochemical cycling (Sinsabaugh and Follstad Shah, 2012). Extracellular enzymes are generated by individual cells, but are retained within the EPS matrix for use by the community of microbes enclosed within the biofilm (Flemming and Wingender, 2010; Flemming et al., 2016). By investigating the relative activities of extracellular enzymes catalyzing the terminal reactions targeting specific resources (i.e., C, N, and P), it is possible to deduce where biofilm energy and nutrient acquisition effort is directed (Sinsabaugh et al., 2009), providing important insights into the ecology of GFSs.

In this study, we investigated how GFS biofilms meet C and nutrient demands through their extracellular enzymatic activity (EEA), and how rates of elemental acquisition may be altered following shifts in streamwater solute concentration and turbidity accompanying the “peak water” transition. Specifically, we ask: (1) how does extracellular enzyme activity and biofilm biomass change over a gradient in glacier influence, and (2) are extracellular enzyme activities related to corresponding inorganic resources and biofilm characteristics? Given the low availability of resources in most GFSs, we hypothesized that resource acquisition through extracellular enzymes may be prioritized, and hence we expect higher acquisition effort to correspond with lower resource availability and increasing glacial influence. To address these questions, we sampled benthic sediments from 20 GFSs within the New Zealand’s Southern Alps, and measured their extracellular enzyme activities along with corresponding biofilm and water column characteristics.

## Materials and Methods

### Study Sites

**FIGURE 1:**
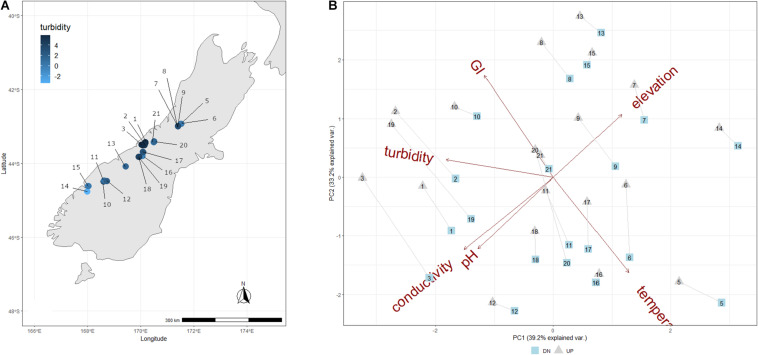
**(A)** Map showing the location of each sample site, and colored according to the corresponding log-transformed turbidity value measured for the overlying streamwater. **(B)** PCA of abiotic variables measured for each New Zealand stream and transect. Upper transects are indicated as gray triangles, and down transects are indicated as blue squares, and transects from a given stream are connected by a gray line.

The GFS catchments are located within three major geological formations on a 340 km transect along the Southern Alps. These include the Greywacke basement terranes of the eastern Alps, the Haast schists to the west, and granite gneisses of Fjordland (Chinn et al., 2014). In total, 20 GFSs were sampled (January–March 2019), and were numbered from GL01 to GL21 for brevity (with GL04 omitted due to error) (Table 1 and Figure 1A). Briefly, Franz Josef, Victoria, and Fox glaciers (GL01–GL03) were sampled from the West Coast region. Lancelot, Crow, White, Marmaduke Dixon, and Cahill glaciers (GL05–GL09) were sampled from Arthur’s Pass. Dart, Reid, and Rob Roy glaciers (GL10–GL12) were sampled from the Wanaka region. Brewster glacier (GL13) was sampled from Haast Pass, and McPherson and Age glaciers (GL14 and GL15) from Milford Sound. Birch Hill, Stocking, Charity, and Richardson glaciers (GL16–GL19) were sampled from the Mt. Cook region. Lastly, Mawson and Shackleton glaciers (GL20 and GL21) were sampled from the Ice Lake region.

**TABLE 1:** Abbreviations, names, GPS coordinates, and physical characteristics of sampled glacier streams.

Code	Glacier name	Glacier surface area (km^2^)	Location	Distance to glacier (m)	Glacial index (GI)	Latitude	Longitude	Altitude (m a.s.l.)	Temperature (°C)	DO (mg L^–1^)	pH	Conductivity (μS cm^–1^)	Turbidity (NTU)
GL01	Franz Josef	35.53	UP	397.0	0.938	−43.4517	170.1734	354	2.4	13.30	7.8	60.1	363.0
			DN	2,907.0	0.672	−43.4274	170.1734	210	3.1	13.40	7.7	49.4	347.7
GL02	Victoria	3.93	UP	55.5	0.973	−43.4976	170.1390	1,186	0.0	12.80	9.1	64.0	227.0
			DN	854.7	0.699	−43.4978	170.1291	1,100	1.3	12.75	8.3	78.8	164.3
GL03	Fox	40.18	UP	627.5	0.910	−43.5020	170.0597	266	0.3	15.38	8.8	77.2	265.3
			DN	3,468.8	0.646	−43.4869	170.0293	219	4.3	12.72	8.3	109.2	352.7
GL05	Lancelot	0.02	UP	820.5	0.147	−42.9249	171.5141	1,326	7.7	10.35	7.5	32.4	0.1
			DN	987.3	0.125	−42.9263	171.5149	1,264	9.6	9.89	7.6	32.5	<0.1
GL06	Crow	0.51	UP	461.9	0.607	−42.9241	171.5137	1,362	8.0	10.31	7.6	9.2	7.8
			DN	1,293.5	0.356	−42.9307	171.5185	1,117	8.0	10.49	8.2	15.0	0.9
GL07	White	0.31	UP	317.9	0.637	−42.9984	171.3898	1,779	3.0	11.04	6.7	5.9	37.7
			DN	583.4	0.488	−42.9963	171.3913	1,707	3.6	10.98	6.8	6.3	32.3
GL08	Marmaduke Dixon	0.63	UP	15.6	0.981	−42.9875	171.3904	1,670	0.7	11.86	7.3	14.9	12.6
			DN	199.4	0.799	−42.9884	171.3923	1,604	1.3	11.74	7.2	14.2	11.1
GL09	Cahill	0.61	UP	180.0	0.813	−42.9837	171.3989	1,391	3.6	11.51	7.2	12.5	23.1
			DN	593.3	0.568	−42.9840	171.4028	1,246	6.0	10.99	6.8	12.2	27.2
GL10	Dart	8.62	UP	12.2	0.996	−44.4813	168.6059	1,111	0.7	13.16	8.1	40.7	49.0
			DN	650.0	0.819	−44.4818	168.5979	1,077	0.7	12.92	7.4	40.8	98.6
GL11	Reid	0.42	UP	320.0	0.669	−44.4673	168.6216	1,581	2.9	11.14	10.2	14.5	4.1
			DN	571.1	0.532	−44.4689	168.6193	1,474	5.4	10.65	10.2	19.0	1.9
GL12	Rob Roy	1.79	UP	963.3	0.581	−44.4758	168.7268	756	6.6	11.35	9.8	66.7	9.9
			DN	1,466.2	0.477	−44.4803	168.7265	714	7.0	11.39	9.8	41.7	8.2
GL13	Brewster	1.80	UP	17.7	0.987	−44.0819	169.4317	1,720	0.7	11.58	6.8	9.3	4.9
			DN	240.5	0.848	−44.0838	169.4305	1,662	0.9	11.93	6.7	6.9	4.5
GL14	McPherson	0.30	UP	309.2	0.639	−44.7565	167.9857	1,079	3.4	11.81	6.4	4.5	<0.1
			DN	402.3	0.577	−44.7571	167.9865	1,056	4.9	11.37	6.2	4.5	<0.1
GL15	Age	1.42	UP	26.5	0.978	−44.6128	168.0224	1,281	1.4	11.94	6.5	10.4	0.8
			DN	57.0	0.954	−44.6114	168.0244	1,245	1.6	12.04	6.6	10.5	1.2
GL16	Birch Hill	0.15	UP	907.1	0.299	−43.7940	170.0643	1,342	5.7	10.63	7.8	95.8	0.7
			DN	1,164.5	0.250	−43.7946	170.0674	1,209	4.9	11.03	7.8	97.5	0.4
GL17	Stocking	0.47	UP	598.1	0.534	−43.6891	170.0822	1,229	5.9	10.71	7.5	21.2	23.0
			DN	1,144.7	0.375	−43.6936	170.0849	1,007	6.9	10.43	7.6	26.0	16.7
GL18	Charity	0.43	UP	380.8	0.633	−43.8169	169.9252	1,204	4.2	11.35	8.0	119.0	2.2
			DN	650.3	0.502	−43.8184	169.9279	1,117	5.5	11.04	8.0	106.3	11.2
GL19	Richardson	2.45	UP	53.0	0.967	−43.8172	169.9336	1,177	0.1	11.23	8.7	122.4	232.7
			DN	1,179.3	0.570	−43.8239	169.9231	1,094	1.8	11.25	8.3	126.5	65.5
GL20	Mawson	0.75	UP	95.6	0.901	−43.4208	170.4904	1,475	2.6	11.80	7.8	68.4	1.5
			DN	1,275.3	0.404	−43.4074	170.4994	990	5.3	11.43	7.8	62.6	2.3
GL21	Shackleton	2.15	UP	144.5	0.910	−43.4043	170.5064	1,084	3.4	11.99	7.6	33.7	2.3
			DN	620.8	0.703	−43.4054	170.5040	1,014	2.7	12.25	7.5	25.4	4.7

### Sediment Sampling

Between January and March 2019, we sampled three individual patches within a 5 m radius in two reaches from each of the 20 GFSs; one upstream (UP) and close to the glacier terminus, and one downstream (DN) (Table 1). The two reaches were sampled in order to investigate how glacier influence affects biofilm characteristics both within streams, as well as across sites. The UP location was sampled on average 335 m from the glacier terminus (range: 12–963 m; median: 314 m) as assessed from satellite images, and the DN location was sampled on average 1,015 m from the terminus (range: 57–3,469; median: 752.5 m). Sampling sites were often logistically determined given physical barriers that restricted access, and thus the UP and DN locations could not be evenly distributed among all GFSs.

From each patch, the top <5 cm of streambed sediments (grain size ranging from 250 μm to 3.15 mm) were sampled using flame-sterilized scoops and graded sieves. Sediment was transferred to sterile cryovials and immediately flash-frozen in liquid nitrogen for the measurements of EEA, chlorophyll *a* concentration (Chl *a*) and EPS (∼30 g wet sediment for EEA, ∼10 g for each of Chl *a* and EPS). While we acknowledge that fresh sediment is ideal for EEA analyses, freezing is an acceptable preservation method when logistically necessary (Hewins et al., 2016). Lastly, approximately 2.5–3 g of wet sediment for bacterial abundance (BA) was collected in a cryovial and fixed in 1.8 ml filter-sterilized paraformaldehyde/glutaraldehyde solution (1% w/v paraformaldehyde, 0.05% w/w glutaraldehyde; Duarte et al., 2020), flash-frozen in the field, and stored at −80°C until analysis.

### Physical and Chemical Characteristics of Meltwater and Catchments

At each study site, streamwater temperature, pH, electrical conductivity, and O_2_ concentration were measured with a multi-parameter probe (MultiLine^®^ Multi 3630 IDS, WTW, Germany). Streamwater turbidity was measured in nephelometric turbidity units (NTU) with a portable turbidity meter (Turb^®^ 430 IR, WTW). Samples for dissolved nutrients and DOC were taken only at the UP sites, and were collected by filtering meltwater through pre-ashed GF/F filters (Whatman, United Kingdom). Samples for DOC were stored in acid-washed and combusted glass vials in the dark and at 4°C, while samples for the analysis of dissolved nutrients were stored in acid-washed 30 ml Nalgene HDPE bottles and frozen at −20°C upon return to the base camp. Aliquots for nutrient analyses were analyzed as in Kohler et al. (2020) using a LaChat QuikChem 8500 flow injection analyser for nitrite/nitrate (NO_2_^–^ + NO_3_^–^, QuikChem Method 31-107-04-1-K), ammonium (NH_4_^+^, Method 31-107-06-1-I), and soluble reactive phosphorus (SRP, Method 31-115-01-1-I). Samples for the analysis of major cation and anions were filtered through 0.2 μm filters, stored at 4°C and measured with a Metrohm 930 Compact IC flex system. DOC concentration was determined on a Sievers M9 TOC Analyser (GE, Boston, MA, United States).

Among GFSs, the distance of a particular GFS site to the glacier terminus, along with the size of the glacier, will effectively determine many of the influential components of the habitat template (Mellor et al., 2017). To account for the collective influence of these related variables on biofilm characteristics, glacier area and the distance to the terminus were measured using satellite data. Specifically, glacier area was defined as the total glacierized area within the catchment above the upper sampling point and was manually delineated from Sentinel 2 imagery (Level 2a, March–April 2019, downloaded from)^1^ based on a catchment definition derived from the ASTER Global Digital Elevation Model (GDEM) Version 3 (NASA/Meti/Aist/Japan Spacesystems, and U. S. /Japan Aster Science Team, 2019). Straight-line distances to the termini were calculated from manually mapped terminus positions based on the same data sources and GPS coordinates of the sampling points measured with a handheld GPS device (GPSMAP^®^ 66s, GARMIN). We then calculated a “Glacial Index” (hereafter abbreviated to “GI”) as defined by Jacobsen and Dangles (2012):

$$\text{GI}=\frac{\sqrt{\mathrm{glacier}\mathrm{area}\left({\text{km}}^{2}\right)}}{\mathrm{distance}\mathrm{from}\mathrm{terminus}\left(\text{km}\right)+\sqrt{\mathrm{glacier}\mathrm{area}\left({\text{km}}^{2}\right)}}$$

Thus, sites with larger GI values are expected to be under greater glacial influence than sites with lower values.

### Extracellular Enzyme Activity

Extracellular enzyme activities were quantified by measuring potential activities of α-1,4-glucosidase (AG), β-1,4-glucosidase (BG), leucine aminopeptidase (LAP), β-1,4-N-acetylglucosaminidase (NAG), and acid (alkaline) phosphatase (AP) using fluorescent 4-Methylumbelliferone (MUF) and 7-Amino-4-methylcoumarin (AMC)-linked substrates (4-MUF-α-D-glucoside, 4-MUF- β -D-glucoside, L-Leucine-7-amino-4-AMC, 4-MUF-N-acetyl-β-D-glucosaminide, and 4-MUF-phosphate, respectively). These enzymes were chosen based upon their function in nutrient acquisition, and widespread use and application in ecological stoichiometry literature (e.g., Sinsabaugh et al., 2009, Sinsabaugh and Follstad Shah, 2012). Briefly, AG and BG are C-acquiring enzymes, primarily degrading starch and cellulose, respectively (Sinsabaugh et al., 2008, 2009; German et al., 2011). Of the N-acquiring enzymes, LAP degrades peptides, and NAG decomposes chitin and peptidoglycan (e.g., fungal and bacterial cell walls) (Sinsabaugh et al., 2009; German et al., 2011). Lastly, AP is a P-acquiring enzyme that liberates phosphate from phosphomonoesters (Sinsabaugh et al., 2008).

Pilot studies on sediments from GFSs in the Swiss Alps served to determine substrate-saturating concentrations through generating saturation curves and the duration of linearity between incubation time and product generation (German et al., 2011). Based on these results, a substrate concentration of 0.3 μM and a 1.5–2 h incubation time were applied to all substrates; this is in agreement with studies from similar ecosystems utilizing comparable methods (e.g., Pastor et al., 2019). Artificial substrates were dissolved in artificial streamwater (e.g., German et al., 2011; Freixa et al., 2017) modeled after the cation/anion composition of published GFS data (from Brown, 2002; McKnight et al., 2004; Singh et al., 2017; Kohler et al., 2020). Briefly, artificial water was made by adding 6.04 mg CaCl, 1 mg KCl, 9 mg MgSO_4_, and 20 mg NaHCO_3_ to 1 L milliQ water. The pH was adjusted to 7.5 with small amounts of HCl (or NaOH if necessary), and autoclaved to ensure that the liquid is enzyme-free.

Sediments were thawed and approximately 1 g (wet weight) was transferred to 15 ml centrifuge tubes. Four ml of MUF and AMC-linked substrates (at +4°C) were added to sediments at a concentration of 0.3 μM (minimum final concentration, taking into consideration the water content of sediment) and the sediments were subsequently incubated for 1.5–2 h in the dark on a shaker at +4°C to mimic the average GFS streamwater temperature. Additionally, reference standards of MUF/AMC were incubated with several controls for each sediment sample, and included a blank (artificial streamwater only), a matrix control (sediment plus artificial streamwater), a quench control (sediment plus MUF/AMC reference standard), and a deactivated control (sediment boiled for 30 min before substrate addition). These controls allowed the quantification of the background fluorescence of artificial streamwater and sediment, as well as potential signal attenuation (i.e., quenching), and the background fluorescence and abiotic degradation of the artificial substrate, respectively.

Following incubation, 2 ml of glycine buffer (pH = 10.4) was quickly added to all tubes (2:1 sample:buffer vol:vol ratio) to raise pH and halt product generation. Tubes were vortexed briefly, centrifuged (at 3,234 *g* for 3 min at 4°C), and 0.2 ml of the supernatant transferred to black 96-well plates (Corning, flat bottom, polystyrene non-binding surface). Fluorescence was read on a BioTek Synergy H1 high sensitivity plate reader at 365/455 excitation/emission wavelengths for MUF and 364/445 excitation/emission wavelengths for AMC. Following analyses, sediments were oven-dried to a stable dry mass (DM) and weighed, and enzymatic activities (nmol hr^–1^ g^–1^ DM) were calculated using equations adapted from DeForest (2009) and German et al. (2011):

$$\begin{array}{rc} & \mathrm{EEA}=\;\\ & \frac{\mathrm{Net}\;\mathrm{fluorescence}\times \mathrm{Artificial}\;\mathrm{streamwater}\;\mathrm{volume}\;\mathrm{in}\;\mathrm{tube}\;(\mathrm{ml})}{\begin{array}{l} \mathrm{Emission}\;\mathrm{coefficient}\times \mathrm{volume}\;\mathrm{in}\;\mathrm{well}\;\left(\mathrm{ml}\right)\times \\ \mathrm{incubation}\;\mathrm{time}\;\left(h\right)\times \mathrm{dry}\;\mathrm{mass}\;\mathrm{sediment}\;(g) \end{array}} \end{array}$$

where:

$$\begin{array}{rc} & \mathrm{Emission}\;\mathrm{coefficient}=\;\\ & \frac{\mathrm{Reference}\;\mathrm{standard}\;\mathrm{fluorescence}-\mathrm{Blank}\;\mathrm{fluorescence}}{\mathrm{Reference}\;\mathrm{standard}\;\mathrm{in}\;\mathrm{well}\;\left(\mathrm{nmol}\right)\;} \end{array}$$

$$\begin{array}{rc} & \mathrm{Net}\;\mathrm{fluorescence}=\;\\ & \frac{\mathrm{Assay}\;\mathrm{fluorescence}-\mathrm{Deactivated}\;\mathrm{control}\;\mathrm{fluorescence}}{\mathrm{Quench}\;\mathrm{coefficient}} \end{array}$$

and:

$$\begin{array}{rc} & \mathrm{Quench}\;\mathrm{coefficient}=\\ & \frac{\mathrm{Quench}\;\mathrm{control}\;\mathrm{fluorescence}-\mathrm{Matrix}\;\mathrm{control}\;\mathrm{fluorescence}}{\mathrm{Reference}\;\mathrm{standard}\;\mathrm{fluorescence}} \end{array}$$

### Biofilm Biomass and EPS

Sediment Chl *a* was measured fluorometrically after an over-night extraction in EtOH (Battin et al., 2001). Briefly, 5 ml of 90% EtOH was added to ca. 2 g of wet sediment, heated to 78°C for 10 min, and incubated in the dark at 4°C for 24 h. Samples were vortexed, centrifuged, and 0.2 ml aliquots were read in a plate reader at 436/680 nm excitation/emission. Concentrations were quantified by comparing against a standard curve of spinach standard, and are reported as μg Chl *a* g^–1^ DM.

Extracellular polymeric substances were quantified as in Battin et al. (2001, 2004) and Romaní et al. (2008). Roughly 10 g of sediment was lyophilized and weighed (DM). Then, 15 ml of 50 mM EDTA was added and samples placed on a rotary shaker at room temperature for 1 h. Samples were centrifuged at 3,234 *g* for 20 min at 4°C. The supernatant was filtered through 0.2 μm to remove particles and bacterial cells, 35 mL ice-cold 99% EtOH was added (70% final concentration), and tubes were incubated at −20°C for 24 h. Samples were centrifuged again for 10 min at 4°C to precipitate the pellet and the supernatant was discarded. The pellet was re-suspended in 5 mL MQ water, 11.7 mL ice-cold 99% EtOH added (70% final concentration), and incubated a second time at −20°C for 24 h. The samples were centrifuged again at 3,234 *g* for 20 min at 4°C, the supernatant discarded, and the pellet allowed to dry at 60°C before being re-suspended in 0.5 mL MQ water. EPS (as bulk carbohydrates) was then measured using the phenol-sulfuric acid method (Dubois et al., 1956). Briefly, 12.5 μl of 80% phenol was added to the extract, followed by 1.25 ml of concentrated (95–97%) sulfuric acid. Samples were kept at room temperature for 10 min, then transferred to ice for 20 min. Samples and a standard curve of glucose were quantified by measuring absorbance at 490 nm, with the final unit being μg glucose equivalents g^–1^ DM.

Bacterial abundances was quantified using flow cytometry following the detachment of cells from the sediment matrix (Amalfitano and Fazi, 2008; Besemer et al., 2009). To detach cells, thawed samples underwent two rounds of mild shaking (Standard Analog Shaker, VWR, 15 min, 5.5 speed) followed by sonication (Sonifier 450, Branson, 1 min, 60% duty cycle, output 5) in 10 ml of the paraformaldehyde/glutaraldehyde solution described above supplemented with sodium pyrophosphate at a final concentration of 0.025 mM. We pooled the resulting extracts per sample (∼20–22 ml total), mixed them thoroughly, transferred 1 ml of each in a sterile 1.5 ml tube, and centrifuged them for 5 s to pellet the large sediment particles. We then diluted 100 μl of the supernatants 10-fold in paraformaldehyde/glutaraldehyde solution and stained the dilutions with SybrGreen^®^ (1× final concentration, incubation for 15 min at 37°C) before analyzing them on a NovoCyte flow cytometer (ACEA Biosciences) equipped with a 488 nm laser. To analyze the stained samples we set the reading time to 2 min per sample and the flow rate to 14 μl per min, rinsing thrice and shaking once between samples. We identified and gated the cell populations based on the height of their fluorescence signals on a 530/30–725/40 nm biplot as previously described (Hammes et al., 2008) using the ACEA NovoExpress^®^ software with thresholds of 300 and 3,000 on the front scatter and 530/30 nm channels, respectively. We analyzed three stained technical replicates plus one unstained replicate of the same extract per sample, the latter to exclude any background fluorescence and to examine for possible autofluorescent populations (e.g., from pigmented cells). The coefficient of variation among technical replicates was 7.5 ± 5.1% on average. Finally, cell numbers were corrected for the various dilution factors and sediment water content to obtain total cells g^–1^ DM.

### Statistical Analyses

Distributions of all variables were first assessed using histograms, and were log-transformed if needed to obtain approximately normal distributions. A constant of 0.65× the value of the smallest non-zero datum was applied to each analyte if necessary to allow log-transformation of zeros resulting from variables below the limit of detection. To explore environmental gradients present among GFS sites, as well as the relationships of these variables to each other, we used Principal Component Analysis (PCA) including GI, turbidity, elevation, conductivity, pH, and water temperature of all transects using *ggbiplot* (Vu, 2011), with all variables mean-centered and standardized.

To normalize EEA by cell number, replicates were averaged by patch (creating three values per glacier UP and DN site, respectively) and normalized by dividing by the average corresponding BA. To characterize nutrient acquisition effort through enzymatic activity, nutrient ratios were created by summing enzyme activities related to acquiring C (AG + BG), N (NAG + LAP), and P (AP), and creating enzymatic C:N, C:P, and N:P ratios with the resulting values. Nutrient limitation based on EEA was inferred by comparing EEA ratios to 1:1 lines indicating equal acquisition effort (Sinsabaugh et al., 2009).

To investigate our main question of how biofilm characteristics will change as glaciers shrink, we utilized GI and turbidity as proxies for glacial influence – the former because it directly approximates changes in glacial volume and proximity, and the latter because it provides an important mechanism of influence on GFS biofilms. Individual pairwise associations were assessed using linear models (lm) in R (R Core Team, 2017), and adjusted *R*^2^ values are reported. The full dataset was used to compare EEA with biofilm characteristics, GI, and turbidity, but since streamwater chemistry was only characterized for UP sites, comparisons between EEA and DOC/nutrients were limited to this subset of the data only.

## Results and Discussion

### Physical and Chemical Characteristics of Streamwater

The sampled GFSs varied considerably in streamwater characteristics, reflecting a gradient from turbid and cold, with low streamwater electrical conductivity and pH values, to relatively clear streams with elevated streamwater temperatures and conductivity (Table 1). Turbidity ranged from 0 to 363 NTU, streamwater temperature from 0 to 9.4°C, electrical conductivity from 4.5 to 126.5 μS cm^–1^, and pH from 6.2 to 10.2. The first two PC axes explained 72.4% of variation among these parameters, and in general, UP and DN sites from the same GFSs clustered together, reflecting their overall similarity in physico-chemical characteristics (Figure 1B). Lower temperatures and greater turbidity were associated with greater GI, and lower pH and conductivity values were found at higher elevations, which may reflect differences in subglacial weathering and the particular climatic conditions of New Zealand where large glaciers nearly reach sea level at the exposed west coast.

Streamwater nutrient and DOC concentrations also differed substantially between GFSs (Table 2). SRP concentrations were low overall, ranging from 5.5 to 13.6 μg P L^–1^, while dissolved inorganic nitrogen (DIN) ranged from 12.5 to 148.2 μg N L^–1^, and DOC ranged from 47.1 to 148.2 μg C L^–1^. In general, GL01, GL02, and GL03 consistently had higher nutrient and DOC concentrations than the other GFSs. Although weak, SRP (*R*^2^ = 0.18, *p* < 0.01), DIN (*R*^2^ = 0.23, *p* < 0.01), and DOC concentrations (*R*^2^ = 0.08, *p* < 0.01) were positively related to streamwater turbidity across the 20 study streams, highlighting the contribution of glacial hydrology and weathering to the delivery of nutrients and carbon to downstream fluvial ecosystems. SRP (*R*^2^ = 0.11, *p* < 0.01) and DIN (*R*^2^ = 0.12, *p* < 0.01) were also positively correlated with GI, but there was no relationship between GI and DOC.

**TABLE 2:** Major ion and nutrient chemistry for glacial streams.

Code	SRP (ppb)	NO_2_^–^ + NO_3_^–^ (ppb)	NH_4_^+^ (ppb)	DIN (ppb)	DOC (ppb)	Na^+^ (ppb)	Mg^2+^ (ppb)	K^+^ (ppb)	Ca^2+^ (ppb)	Cl^–^ (ppb)	SO_4_^2–^ (ppb)
GL01UP	13.6	44.2	32.1	76.3	143.7	697	350	1,394	7,340	379	4,489
GL02UP	8.2	51.9	34.5	86.3	128.2	642	265	1,984	8,505	283	6,331
GL03UP	7.8	62.2	33.3	95.5	148.2	1,129	544	1,981	10,222	495	9,379
GL05UP	7.3	10.0	25.5	35.4	85.5	671	75	135	4,814	377	1,529
GL06UP	7.2	14.8	24.9	39.7	75.7	161	<LOD	84	1,138	110	371
GL07UP	7.2	23.0	23.1	46.1	121.3	107	<LOD	108	669	67	100
GL08UP	8.0	55.8	24.9	80.7	47.1	340	<LOD	98	1,870	161	988
GL09UP	7.1	41.0	24.5	65.4	143.7	207	<LOD	181	1,579	133	570
GL10UP	6.5	22.2	24.6	46.8	92.4	121	106	180	6,151	60	5,955
GL11UP	6.1	7.6	27.3	34.9	89.2	<LOD	<LOD	61	2,138	51	1,362
GL12UP	6.2	21.3	1.5	22.8	98.3	805	442	746	8,837	146	11,071
GL13UP	6.8	9.6	2.9	12.5	73.6	89	<LOD	49	988	66	987
GL14UP	5.8	8.2	25.4	33.6	108.8	252	<LOD	18	116	265	239
GL15UP	9.1	37.7	24.6	62.3	82.1	521	117	77	574	269	1,688
GL16UP	5.5	17.3	20.2	37.5	127.9	1,539	573	485	14,541	164	19,489
GL17UP	5.6	8.5	23.8	32.3	90.7	192	69	150	4,348	123	991
GL18UP	6.5	35.7	29.1	64.9	59.3	630	373	727	19,557	204	20,277
GL19UP	6.8	45.9	58.1	104.0	66.8	1,093	936	888	18,670	181	26,759
GL20UP	6.2	26.9	29.2	56.1	68.0	699	164	312	10,434	175	8,830
GL21UP	5.6	12.9	21.9	34.8	104.8	787	96	273	5,057	221	3,817

*All concentrations are averaged from duplicate measurements and reported in ppb. “<LOD” indicates that values fell below the limit of detection, which were 19 ppb for Na^+^ and 10 ppb for Mg^2+^.*

### Biomass and Extracellular Enzyme Activity Across the Environmental Gradient

**FIGURE 2:**
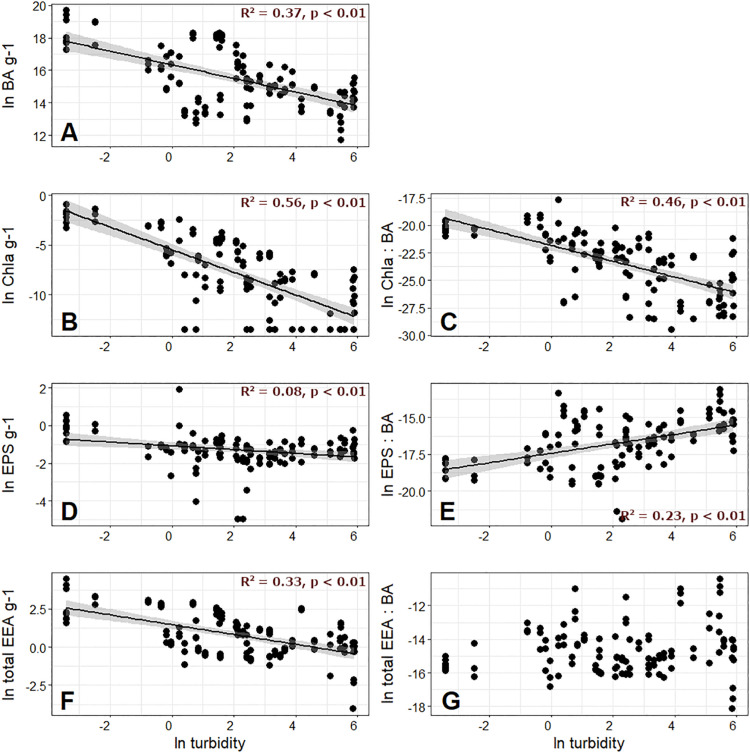
Bacterial abundance (BA) per gram of sediment dry mass, along with measured chlorophyll *a* (Chl *a*) on the second row, extracellular polymeric substances (EPS) on the third row, and total extracellular enzyme activity (EEA) on the fourth row plotted against meltwater turbidity. The left column of the latter variables are given per gram **(A**,**B**,**D**,**F)**, and the right column variables are normalized by BA **(C,E,G)**.

Bacterial abundances ranged from 1.22 × 10^5^ to 3.59 × 10^8^ cells g^–1^ DM and sediment Chl *a* ranged from 0 to 0.41 μg Chl *a* g^–1^ DM (Supplementary Table 1). Compared to streams from other ecotypes (e.g., Romaní and Sabater, 2001; Amalfitano and Fazi, 2008), such low BA and Chl *a* values may reflect the harsh environmental conditions present in GFSs. In support of this notion, when BA and Chl *a* were compared with streamwater turbidity, both were significantly and negatively related (Figures 2A,B), and indicate that conditions may be somewhat more relaxed in the less turbid GFSs draining from smaller glaciers. Meanwhile, EPS ranged from 0 to 6.80 glucose-equivalent g^–1^ DM, and showed a weaker, negative relationship with turbidity (Figure 2D). Finally, BA and Chl *a* were also negatively related to GI, but *R*^2^ values were lower than with turbidity, and there was no significant association with EPS (Supplementary Figures 1A,B,D).

When normalized by BA to create “cell-specific” values, Chl *a* maintained its negative relationship with both turbidity and GI (Figure 2C and Supplementary Figure 1C), while EPS showed a significant, positive relationship with both turbidity and GI (Figure 2E and Supplementary Figure 1E). The greater cell-specific EPS with higher streamwater turbidity is in line with previous work by Battin et al. (2001), and perhaps reflects biofilm strategies to thrive in GFSs where sediments are unstable (Miller and Lane, 2019). Battin et al. (2001) hypothesized that the elevated EPS generated per cell near the glacier terminus may be a strategy for immobilizing and storing allochthonous organic C transported by meltwater, which may be important given diel and seasonal fluctuations in the concentration and lability of this resource. Given that streamwater turbidity overall explained more variability in biofilm biomass characteristics than GI (Figure 2 versus Supplementary Figure 1), and is also predicted to be an important component of the physical template to shift following the “peak water” transition (Milner et al., 2017; Delaney et al., 2018), we retained turbidity as a proxy for glacier influence in subsequent visualizations.

Total EEA ranged from 9.79 × 10^–4^ to 86 nmol g^–1^ DM h^–1^, and were comparable in magnitude to previous studies reporting from desert soils (Stursova et al., 2006; Petrie et al., 2015), seafloor sediments (Coolen and Overmann, 2000; Meyers et al., 2014), and other glacierized catchments (Freimann et al., 2013a, b; Pastor et al., 2019). Like for BA and Chl *a*, total EEA was negatively related to streamwater turbidity (Figure 2F) and GI (Supplementary Figure 1F), and was overall dominated by LAP, accounting for 0 to 100% of the total activity across all samples (Supplementary Table 1). This was followed by AP, which ranged from 0 to 74% of the total activity. Activity rates of BG, AG, and NAG were all considerably lower, resulting in low enzymatic C:N (ranging from 3.0 × 10^–6^ to 0.62) and C:P values (from 9.36 × 10^–5^ to 1.25), and overall high N:P (up to 562).

In order to estimate cell-specific resource acquisition effort, we also normalized EEA by BA. Interestingly, when the resulting total EEA per cell was compared against turbidity and GI, no significant relationships were found (Figure 2G and Supplementary Figure 1G). Given the costly nature of extracellular enzyme generation, which presumably trades off with productivity (Malik et al., 2019, 2020; Ramin and Allison, 2019), we speculate that the reason for this non-relationship may reflect the delicate balance between resource supply, acquisition, and growth in GFSs. Enzyme production is expected to scale with substrate availability such that enzymes operate at about half their maximum catalytic capacity (Sinsabaugh and Follstad Shah, 2012). Exceeding this threshold by producing additional enzyme would reduce the reaction velocity per enzyme, and given the costs for enzyme production would not be an effective strategy. In ultra-oligotrophic systems such as GFSs, biofilms may particularly well tune the metabolic efforts directed toward substrate acquisition.

**FIGURE 3:**
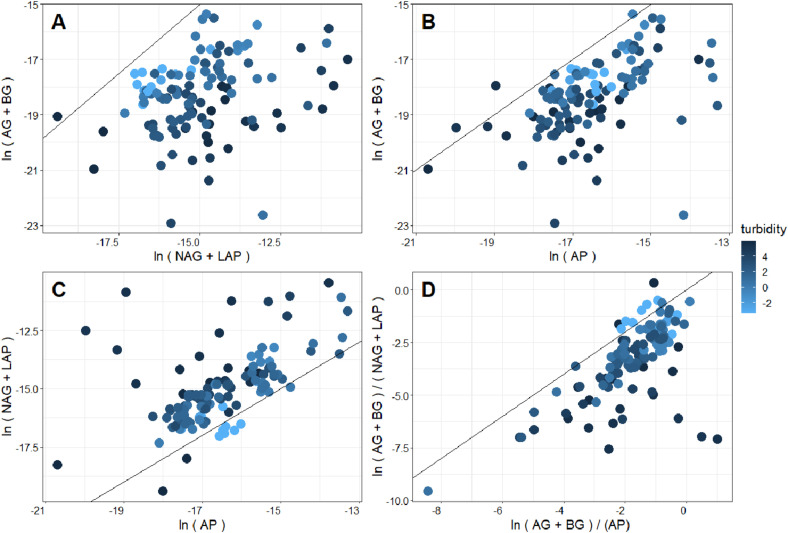
Comparison **(A)** C- versus N-acquiring enzymes, **(B)** C- versus P-acquiring enzymes, **(C)** N- versus P-acquiring enzymes, and **(D)** enzymatic C:N versus C:P stoichiometry of New Zealand glacier meltwater streams. Fill color corresponds to the log-transformed turbidity value of the corresponding meltwater. A 1:1 line is superimposed, which indicates equal acquisition effort by the compared enzymatic activity on the x and y axis (Sinsabaugh et al., 2009). Thus, deviations from this line suggest limitation in favor of the enzyme class where more acquisition effort is directed.

Because microorganisms allocate resources to substrate acquisition depending on substrate availability, pairwise comparisons of EEA can indirectly inform us on substrate limitation. Comparing total C (AG + BG) versus N (LAP + NAG) and P (AP) acquiring EEA showed that generally more microbial effort is directed toward the acquisition of N and P as compared to C, indicating that communities were generally nutrient limited, with the majority of the GFSs being N-limited (Figures 3A–D), which agrees well with past predictions for GFS microbial communities (Ren et al., 2019; Elser et al., 2020). High rates of weathering are hypothesized to take place beneath glaciers, which has the potential to liberate large quantities of P. However, much of this P is sediment-bound (Hawkings et al., 2016), and only a minority is thought to be bioavailable (only ∼1% by one estimate, Hodson et al., 2004). With the exception of GFSs from the Milford Sound region, where most of the P-limited communities were sampled, the bedrock underlying the sampled GFSs is primarily readily erodible schist (Chinn et al., 2014), and ample P loads might be expected in the meltwater. Therefore, it is intuitive to assume that microbial biofilms do not expend superfluous energy in producing P-acquiring enzymes if P is readily available. Instead, elevated activities of LAP compared to other enzymes in most samples indicate a substantial investment in N acquisition.

**FIGURE 4:**
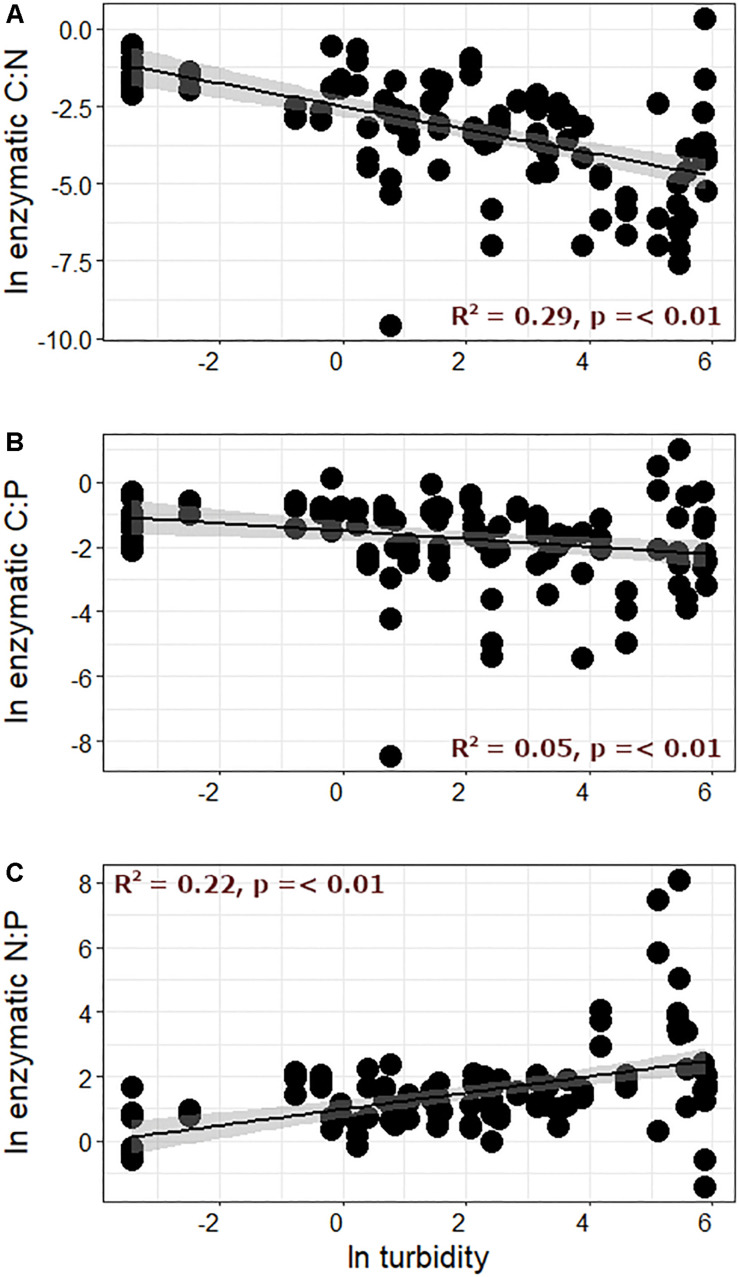
Enzymatic **(A)** C:N, **(B)** C:P, and **(C)** N:P ratios plotted against turbidity.

**FIGURE 5:**
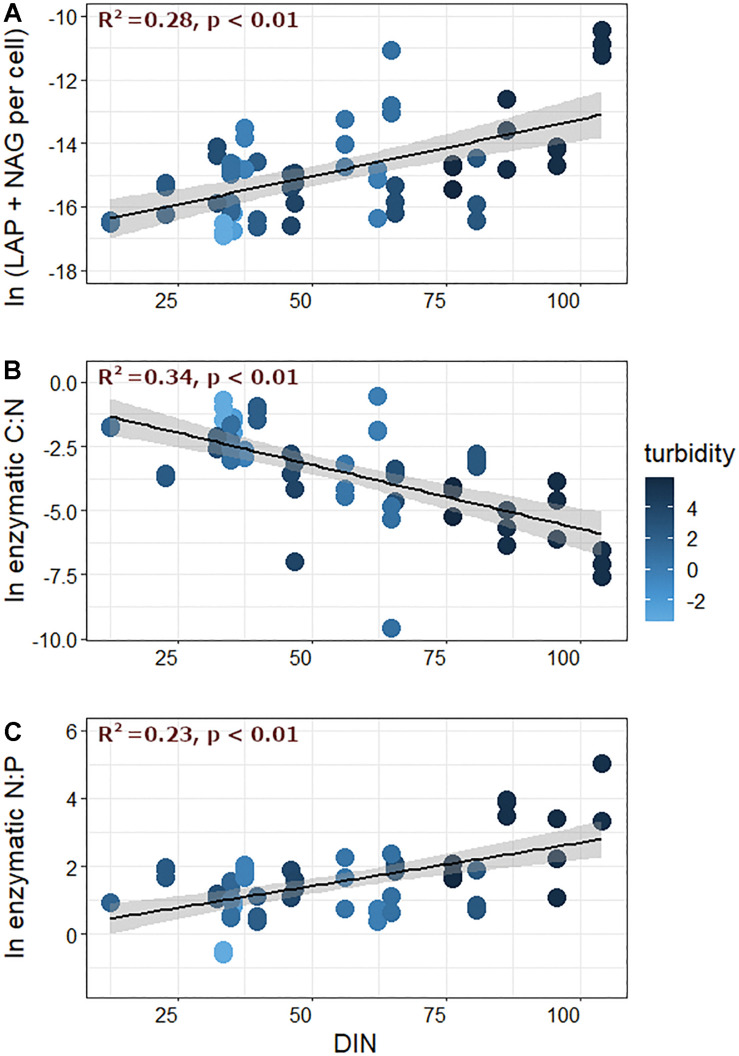
**(A)** N-acquiring enzymes (LAP + NAG), **(B)** enzymatic C:N, and **(C)** enzymatic N:P plotted against dissolved inorganic nitrogen (DIN) concentrations measured in streamwater. Data represent values measured for upper transects only, and fill color corresponds to the log-transformed turbidity value of the corresponding meltwater.

Interestingly, nutrient limitation appeared to intensify (e.g., points increasingly distant from the 1:1 line in Figure 3) as streamwater turbidity increased. When enzymatic C:N:P ratios were directly compared with streamwater turbidity, enzymatic C:N and C:P (Figures 4A,B) significantly decreased with increasing turbidity, while enzymatic N:P increased (Figure 4C). Enzymatic C:N (*R*^2^ = 0.07, *p* < 0.01) and C:P (*R*^2^ = 0.04, *p* = 0.02) were also negatively correlated with GI, but *R*^2^ values were again lower than with turbidity, and enzymatic N:P did not show a significant relationship. Enzymatic C:N:P were further compared against DIN, SRP, and DOC. Enzymatic C:N (Figure 5B) and C:P (*R*^2^ = 0.09, *p* = 0.01) were negatively related to streamwater DIN, while enzymatic N:P (Figure 5C) was positively related. Enzymatic C:N:P ratios were not significantly related to SRP or DOC, but enzymatic C:N was positively correlated with water column DOC:DIN (*R*^2^ = 0.26, *p* < 0.01) and enzymatic N:P positively related to water column DIN:SRP (*R*^2^ = 0.23, *p* < 0.01). Lastly, there was no relationship with enzymatic C:P and water column DOC:SRP.

To gain further insight into possible mechanisms behind these relationships, cell-specific EEA were furthermore compared against turbidity, GI, and nutrient concentrations. Cell-specific C-acquiring EEA was negatively related to turbidity (*R*^2^ = 0.17, *p* < 0.01), but was not correlated with streamwater DIN, DOC, or SRP concentrations. On the other hand, cell-specific N-acquiring EEA was weakly related to turbidity (*R*^2^ = 0.05, *p* < 0.01), not related to DOC or SRP, and was positively correlated with DIN (Figure 5A). Cell-specific P-acquiring EEA exhibited a weakly negative relationship with turbidity (*R*^2^ = 0.04, *p* = 0.02), and showed little/no correlation with DOC or nutrient chemistry. Finally, only C-acquiring enzymes were significantly related to GI, and activities decreased with increasing GI values (*R*^2^ = 0.09, *p* < 0.01).

Given that inorganic forms of nutrients should be readily incorporated by microorganisms, we did not anticipate the strong relationships between N-acquiring EEA and DIN. One possibility is that even though DIN showed variable concentrations among sites, GFS biofilm microbes may have still been N-limited on a mass balance basis. Because organic P and N are generally released together with inorganic forms (Hawkings et al., 2016; Wadham et al., 2016), it may be that P is more abundant than we realized based on the measurements of inorganic concentrations alone. With this same line of reasoning, it may be that more N-acquiring enzymes were produced with increasing DIN because there was a corresponding increase in dissolved organic N. Because we measured only the dissolved inorganic forms of nutrients, there may be much more information to be gained by measuring the many different, potentially bioavailable, fractions (e.g., Hawkings et al., 2016; Wadham et al., 2016), some of which may more bioavailable than we currently appreciate (Mindl et al., 2007).

**FIGURE 6:**
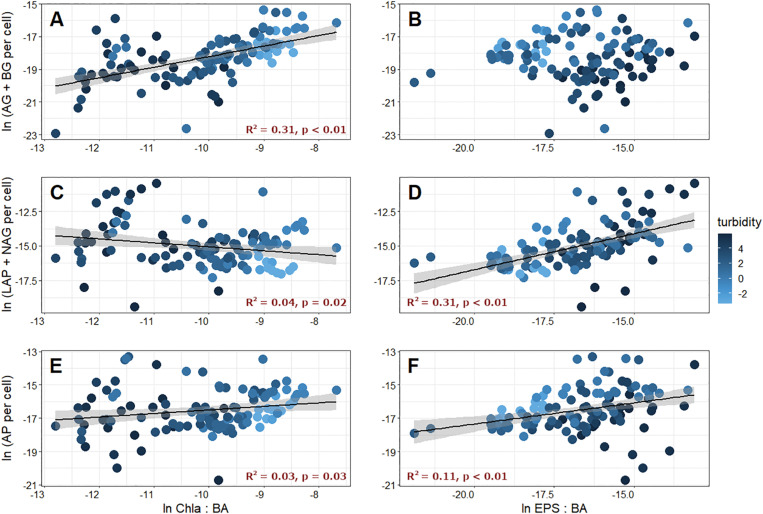
C-acquiring (AG + BG), N-acquiring (LAP + NAG), and P-acquiring (AP) extracellular enzyme activities plotted against corresponding chlorophyll *a* **[**Chl *a*, left column **(A,C,E)]** and extracellular polymeric substances [EPS, right column **(B,D,F)**] normalized by bacterial abundance (BA). Fill color corresponds to the log-transformed turbidity value of the corresponding meltwater.

We also wanted to know how EEA were related to biofilm characteristics, and compared C-, N-, and P-acquiring EEA with cell-specific Chl *a*, which is a measure of the relative importance of photoautotrophy in biofilms. Cell-specific C-acquiring EEA were significantly and positively related to the ratio of Chl *a* and BA (Figure 6A), while N- and P-acquiring EEA were only modestly correlated (Figures 6C,E). As a result, enzymatic C:N (*R*^2^ = 0.39, *p* < 0.01) and C:P (*R*^2^ = 0.15, *p* < 0.01) were positively related to the Chl *a*:BA ratio, and N:P was significantly negatively correlated (*R*^2^ = 0.18, *p* < 0.01). These results are not without precedent, and in previous studies, Espeland et al. (2001) found that both AG and BG were stimulated by photosynthetic activity, and Romaní and Sabater (2000) showed that both BG and AP activities increased with increasing biofilm Chl *a* concentrations. While the association between EEA and Chl *a* is not well understood, it is almost certainly a response to the generation of algal lysis products, which may require enzymes for processing before they can be incorporated into bacterial cells, and may actually induce enzyme production (Espeland et al., 2001). Thus, photoautotrophic activity in GFSs may have a “priming” effect on microbial decomposition by increasing EEA to utilize algal exudates (Battin et al., 2016).

Finally, we found that the total EEA per cell was positively correlated with cell-specific EPS (*R*^2^ = 0.33, *p* < 0.01). Interestingly, cell-specific EPS was positively correlated to cell-specific N-acquiring (Figure 6D) and P-acquiring (Figure 6F) EEA, but was not related to cell-specific C-acquiring EEA (Figure 6B). By extension, enzymatic C:N (*R*^2^ = 0.23, *p* < 0.01) and C:P (*R*^2^ = 0.09, *p* < 0.01) were both significantly and negatively related to cell-specific EPS, while N:P was positively correlated (*R*^2^ = 0.11, *p* < 0.01). Given that the overwhelming majority of GFSs were N (and P)-limited based on comparisons of EEA, it makes sense that more of these enzymes can be retained within an expanded EPS matrix on a per cell basis. However, the other potential roles, and tradeoffs, of EPS including enzyme retention, particle stabilization, and resource storage are poorly explored in GFSs and represent an interesting avenue for further investigation.

## Conclusion

The observed relationships between EEA, Chl *a*, EPS, and turbidity paint an interesting picture for the ecology of GFSs. We found GFS microbial communities to be primarily N-limited, and N-acquiring EEA were positively associated with DIN, while C-acquiring EEA were positively correlated with Chl *a*. We also found strong relationships between biofilm characteristics and streamwater turbidity, highlighting the utility of this variable specifically in explaining patterns among GFSs. Given the efficiency by which fine suspended sediments attenuate light, turbidity places a limit on the accumulation and development of phototrophic microbial biofilms, and photoautotrophy will likely contribute a greater proportion of organic resources to GFSs following the “peak water” transition. Lastly, the greater cell-specific EPS production with higher turbidity may be necessary in heavily glacierized catchments to trap and degrade organic molecules, retain extracellular enzymes, and bind the entire matrix to substrata. Collectively, our results provide some of the first insights into the activity of extracellular enzymes in GFSs, and reveal important biogeochemical patterns which may serve as a first step toward understanding the functional strategies microbes employ in these vanishing environments.

## Data Availability Statement

The original contributions presented in the study are included in the article/Supplementary Material, further inquiries can be directed to the corresponding author.

## Author Contributions

TB, TK, and HP conceived of the project. HP, MT, VS, and MSt performed the fieldwork and collected samples. TK, HP, SF, PP, MT, MSc, and VS performed laboratory work and calculated indices. TK analyzed the data and wrote the manuscript with significant input and editing from all co-authors. All authors contributed to the article and approved the submitted version.

## Conflict of Interest

AW was employed by the company Selva Analytics, LLC. The remaining authors declare that the research was conducted in the absence of any commercial or financial relationships that could be construed as a potential conflict of interest.

## Footnotes

1 https://scihub.copernicus.eu/

## Supplementary Material

Supplementary Table 1 Characteristics for glacier fed stream biofilms. Reported values are averages and standard deviations calculated from the three patches sampled at each transect.
