# Supplementary material for: Patterns and Drivers of Extracellular Enzyme Activity in New Zealand Glacier-Fed Streams
Source: Front Microbiol. 2020 Nov 19;11:591465. doi: 10.3389/fmicb.2020.591465 (PMC7711088; doi:10.3389/fmicb.2020.591465)

**Table S1**

| **Code** | **BA**  **(× 10^5^ g^-1^ DM)** | **Chl *a***  **(ng g^-1^ DM)** | **EPS**  **(µg glucose-** **equivalents g^-1^ DM)** | **AG**  **(nmol g^-1^ h^-1^)** | **BG**  **(nmol g^-1^ h^-1^)** | | | **LAP**  **(nmol g^-1^ h^-1^)** | **NAG**  **(nmol g^-1^ hr^-1^)** | **AP**  **(nmol g^-1^ hr^-1^)** |
| --- | --- | --- | --- | --- | --- | --- | --- | --- | --- | --- |
| GL01UP | 31.46 ± 21.59 | 0.11 ± 0.15 | 0.47 ± 0.28 | 0.006 ± 0.007 | | 0.005 ± 0.003 | 0.877 ± 0.239 | | 0.004 ± 0.007 | 0.146 ± 0.058 |
| GL01DN | 22.74 ± 13.67 | 0.10 ± 0.09 | 0.34 ± 0.15 | 0.004 ± 0.003 | | 0.010 ± 0.009 | 0.667 ± 0.584 | | 0.000 ± 0.000 | 0.107 ± 0.112 |
| GL02UP | 13.57 ± 9.49 | 0.00 ± 0.00 | 0.28 ± 0.10 | 0.003 ± 0.003 | | 0.002 ± 0.002 | 1.345 ± 0.435 | | 0.002 ± 0.002 | 0.031 ± 0.013 |
| GL02DN | 6.98 ± 0.53 | 0.00 ± 0.00 | 0.25 ± 0.06 | 0.004 ± 0.002 | | 0.000 ± 0.002 | 1.194 ± 1.151 | | 0.008 ± 0.007 | 0.020 ± 0.033 |
| GL03UP | 17.15 ± 7.13 | 0.00 ± 0.00 | 0.23 ± 0.01 | 0.005 ± 0.005 | | 0.003 ± 0.003 | 1.031 ± 0.597 | | 0.007 ± 0.009 | 0.156 ± 0.123 |
| GL03DN | 25.14 ± 15.74 | 0.20 ± 0.33 | 0.37 ± 0.10 | 0.004 ± 0.001 | | 0.011 ± 0.006 | 0.191 ± 0.310 | | 0.001 ± 0.002 | 0.096 ± 0.055 |
| GL05UP | 1327.35 ± 778.73 | 157.25 ± 90.15 | 1.35 ± 0.41 | 0.612 ± 0.066 | | 2.213 ± 0.621 | 13.535 ± 4.202 | | 1.111 ± 0.243 | 6.030 ± 1.470 |
| GL05DN | 2781.51 ± 815.38 | 257.70 ± 131.25 | 0.86 ± 0.19 | 1.641 ± 1.318 | | 6.098 ± 1.707 | 41.236 ± 19.871 | | 1.684 ± 0.503 | 13.526 ± 0.912 |
| GL06UP | 240.43 ± 181.71 | 9.17 ± 0.84 | 0.18 ± 0.10 | 0.129 ± 0.033 | | 0.594 ± 0.237 | 2.134 ± 0.317 | | 0.132 ± 0.032 | 1.213 ± 0.453 |
| GL06DN | 150.75 ± 101.34 | 2.36 ± 1.15 | 0.28 ± 0.08 | 0.034 ± 0.007 | | 0.111 ± 0.011 | 0.753 ± 0.126 | | 0.051 ± 0.008 | 0.322 ± 0.032 |
| GL07UP | 52.81 ± 47.29 | 0.20 ± 0.02 | 0.22 ± 0.08 | 0.007 ± 0.002 | | 0.025 ± 0.009 | 0.626 ± 0.091 | | 0.012 ± 0.009 | 0.171 ± 0.059 |
| GL07DN | 30.37 ± 17.91 | 0.18 ± 0.16 | 0.25 ± 0.15 | 0.006 ± 0.001 | | 0.021 ± 0.005 | 0.342 ± 0.063 | | 0.014 ± 0.003 | 0.167 ± 0.028 |
| GL08UP | 27.63 ± 17.28 | 0.10 ± 0.10 | 0.31 ± 0.03 | 0.005 ± 0.001 | | 0.015 ± 0.009 | 0.377 ± 0.110 | | 0.006 ± 0.006 | 0.148 ± 0.106 |
| GL08DN | 43.23 ± 11.68 | 0.17 ± 0.06 | 0.19 ± 0.09 | 0.003 ± 0.001 | | 0.011 ± 0.001 | 0.406 ± 0.065 | | 0.005 ± 0.009 | 0.129 ± 0.016 |
| GL09UP | 27.99 ± 6.66 | 0.01 ± 0.02 | 0.21 ± 0.04 | 0.003 ± 0.001 | | 0.007 ± 0.006 | 0.375 ± 0.107 | | 0.000 ± 0.000 | 0.055 ± 0.020 |
| GL09DN | 33.77 ± 1.48 | 0.05 ± 0.08 | 0.25 ± 0.15 | 0.003 ± 0.001 | | 0.006 ± 0.004 | 0.518 ± 0.103 | | 0.000 ± 0.000 | 0.119 ± 0.028 |
| GL10UP | 52.37 ± 27.90 | 0.22 ± 0.22 | 0.26 ± 0.05 | 0.003 ± 0.003 | | 0.016 ± 0.017 | 1.020 ± 0.174 | | 0.000 ± 0.000 | 0.236 ± 0.071 |
| GL10DN | 31.42 ± 1.28 | 0.25 ± 0.22 | 0.25 ± 0.08 | 0.001 ± 0.001 | | 0.002 ± 0.001 | 1.098 ± 0.314 | | 0.001 ± 0.001 | 0.177 ± 0.076 |
| GL11UP | 739.10 ± 85.95 | 10.44 ± 2.17 | 0.44 ± 0.20 | 0.166 ± 0.032 | | 0.929 ± 0.623 | 7.875 ± 1.541 | | 0.341 ± 0.145 | 1.862 ± 0.148 |
| GL11DN | 799.59 ± 133.95 | 26.12 ± 6.76 | 0.42 ± 0.05 | 0.285 ± 0.136 | | 0.960 ± 0.133 | 13.086 ± 1.194 | | 0.360 ± 0.230 | 3.017 ± 0.657 |
| GL12UP | 140.26 ± 76.15 | 2.74 ± 2.94 | 0.21 ± 0.25 | 0.012 ± 0.011 | | 0.042 ± 0.014 | 2.076 ± 0.296 | | 0.017 ± 0.006 | 0.331 ± 0.046 |
| GL12DN | 130.47 ± 22.82 | 2.83 ± 1.11 | 0.11 ± 0.09 | 0.027 ± 0.013 | | 0.077 ± 0.033 | 2.594 ± 0.886 | | 0.034 ± 0.017 | 0.388 ± 0.078 |
| GL13UP | 698.27 ± 118.05 | 17.23 ± 6.44 | 0.25 ± 0.06 | 0.208 ± 0.071 | | 0.662 ± 0.096 | 4.515 ± 0.909 | | 0.390 ± 0.122 | 1.966 ± 0.357 |
| GL13DN | 641.82 ± 266.64 | 8.25 ± 5.495 | 0.48 ± 0.09 | 0.179 ± 0.037 | | 0.574 ± 0.201 | 4.965 ± 2.799 | | 0.192 ± 0.058 | 1.822 ± 0.183 |
| GL14UP | 575.21 ± 87.96 | 149.96 ± 31.95 | 0.57 ± 0.23 | 0.404 ± 0.410 | | 0.712 ± 0.183 | 2.683 ± 0.045 | | 0.379 ± 0.209 | 5.200 ± 0.313 |
| GL14DN | 442.16 ± 102.75 | 71.12 ± 35.48 | 0.98 ± 0.30 | 0.359 ± 0.420 | | 0.347 ± 0.098 | 1.877 ± 0.400 | | 0.312 ± 0.204 | 3.032 ± 0.499 |
| GL15UP | 89.86 ± 106.80 | 3.28 ± 1.27 | 2.72 ± 3.54 | 0.031 ± 0.010 | | 0.297 ± 0.254 | 1.100 ± 0.484 | | 0.054 ± 0.017 | 0.625 ± 0.170 |
| GL15DN | 96.96 ± 97.56 | 35.72 ± 43.93 | 0.28 ± 0.01 | 0.050 ± 0.028 | | 0.382 ± 0.242 | 1.016 ± 0.073 | | 0.161 ± 0.192 | 1.056 ± 0.462 |
| GL16UP | 206.28 ± 174.36 | 57.75 ± 16.60 | 0.28 ± 0.08 | 0.191 ± 0.039 | | 0.670 ± 0.189 | 12.626 ± 1.752 | | 0.382 ± 0.206 | 2.033 ± 0.321 |
| GL16DN | 130.89 ± 39.33 | 46.55 ± 2.61 | 0.35 ± 0.03 | 0.200 ± 0.051 | | 0.860 ± 0.173 | 15.271 ± 1.043 | | 1.061 ± 1.294 | 2.716 ± 1.027 |
| GL17UP | 62.82 ± 53.67 | 2.66 ± 0.55 | 0.16 ± 0.03 | 0.042 ± 0.001 | | 0.148 ± 0.030 | 1.866 ± 0.363 | | 0.036 ± 0.012 | 0.635 ± 0.070 |
| GL17DN | 58.03 ± 12.07 | 2.24 ± 0.57 | 0.17 ± 0.04 | 0.056 ± 0.001 | | 0.175 ± 0.005 | 2.247 ± 0.114 | | 0.061 ± 0.012 | 0.526 ± 0.036 |
| GL18UP | 4.77 ± 1.59 | 0.12 ± 0.18 | 0.11 ± 0.07 | 0.002 ± 0.003 | | 0.010 ± 0.010 | 2.609 ± 2.290 | | 0.001 ± 0.001 | 0.529 ± 0.102 |
| GL18DN | 6.32 ± 3.48 | 0.03 ± 0.05 | 0.06 ± 0.04 | 0.003 ± 0.004 | | 0.005 ± 0.003 | 1.849 ± 1.429 | | 0.014 ± 0.024 | 0.660 ± 0.024 |
| GL19UP | 2.38 ± 1.20 | 0.00 ± 0.00 | 0.33 ± 0.17 | 0.002 ± 0.002 | | 0.002 ± 0.003 | 4.234 ± 0.666 | | 0.010 ± 0.015 | 0.052 ± 0.065 |
| GL19DN | 10.71 ± 4.33 | 0.00 ± 0.00 | 0.33 ± 0.17 | 0.026 ± 0.014 | | 0.045 ± 0.026 | 11.464 ± 1.011 | | 0.022 ± 0.019 | 0.346 ± 0.184 |
| GL20UP | 6.50 ± 1.08 | 0.11 ± 0.19 | 0.30 ± 0.13 | 0.002 ± 0.001 | | 0.008 ± 0.002 | 0.646 ± 0.476 | | 0.000 ± 0.000 | 0.112 ± 0.010 |
| GL20DN | 7.22 ± 1.70 | 0.43 ± 0.45 | 0.31 ± 0.05 | 0.004 ± 0.001 | | 0.014 ± 0.006 | 0.451 ± 0.093 | | 0.004 ± 0.008 | 0.154 ± 0.027 |
| GL21UP | 14.63 ± 1.64 | 1.88 ± 0.45 | 0.24 ± 0.07 | 0.007 ± 0.001 | | 0.047 ± 0.034 | 0.486 ± 0.032 | | 0.035 ± 0.027 | 0.273 ± 0.063 |
| GL21DN | 13.17 ± 6.74 | 0.11 ± 0.06 | 0.22 ± 0.05 | 0.004 ± 0.001 | | 0.009 ± 0.005 | 0.359 ± 0.120 | | 0.052 ± 0.017 | 0.118 ± 0.031 |

**Figure S1** Bacterial abundance (BA) per gram of sediment dry mass, along with measured chlorophyll *a* (Chl *a*) on the second row, extracellular polymeric substances (EPS) on the third row, and total extracellular enzyme activity (EEA) on the fourth row plotted against the Glacial Index (GI). The left column of the latter variables are given per gram (panels A, B, D, and F), and the right column variables are normalized by BA (panels C, E, and G).

**
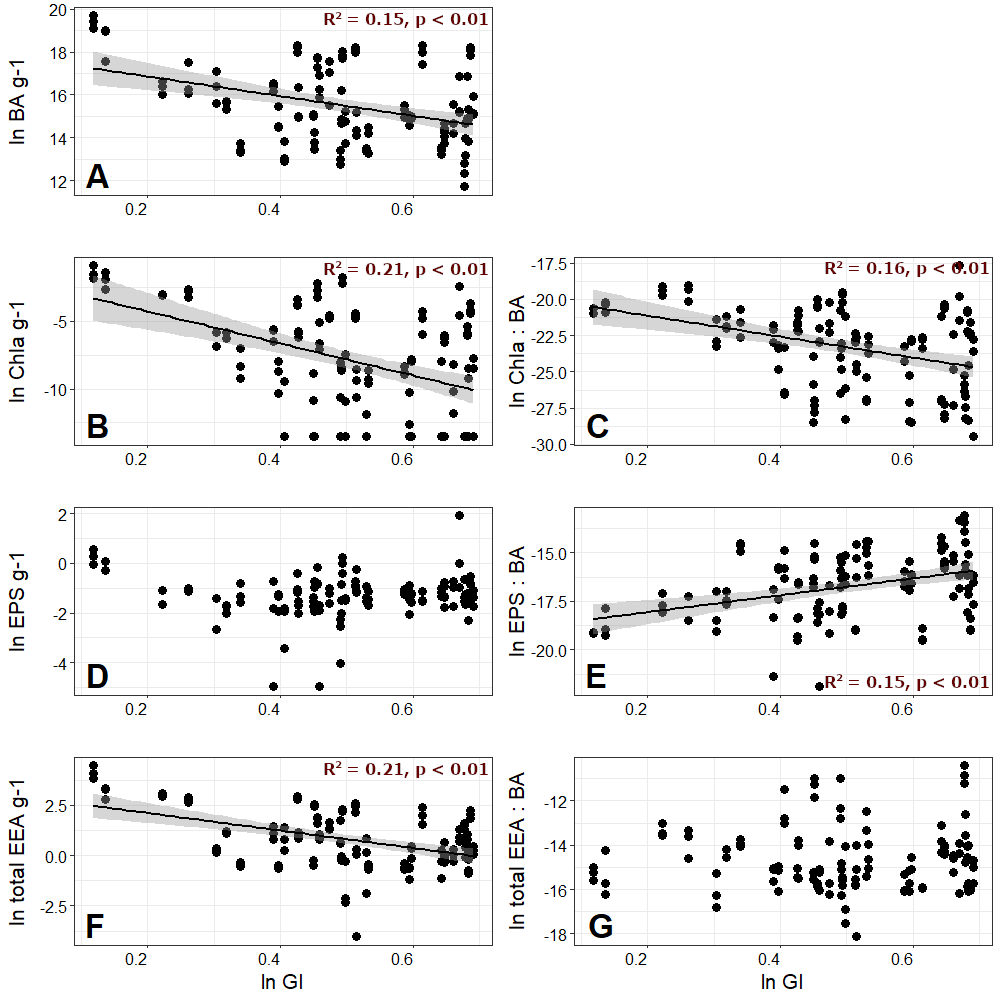
**

**Figure S2** Bacterial abundance (BA) per gram of sediment dry mass, along with measured chlorophyll *a* (Chl *a*) on the second row, extracellular polymeric substances (EPS) on the third row, and total extracellular enzyme activity (EEA) on the fourth row plotted against log-transformed turbidity. The left column of the latter variables are given per gram (panels A, B, D, and F), and the right column variables are normalized by BA (panels C, E, and G). The color of datapoints and trendlines correspond to UP vs. DN transects.


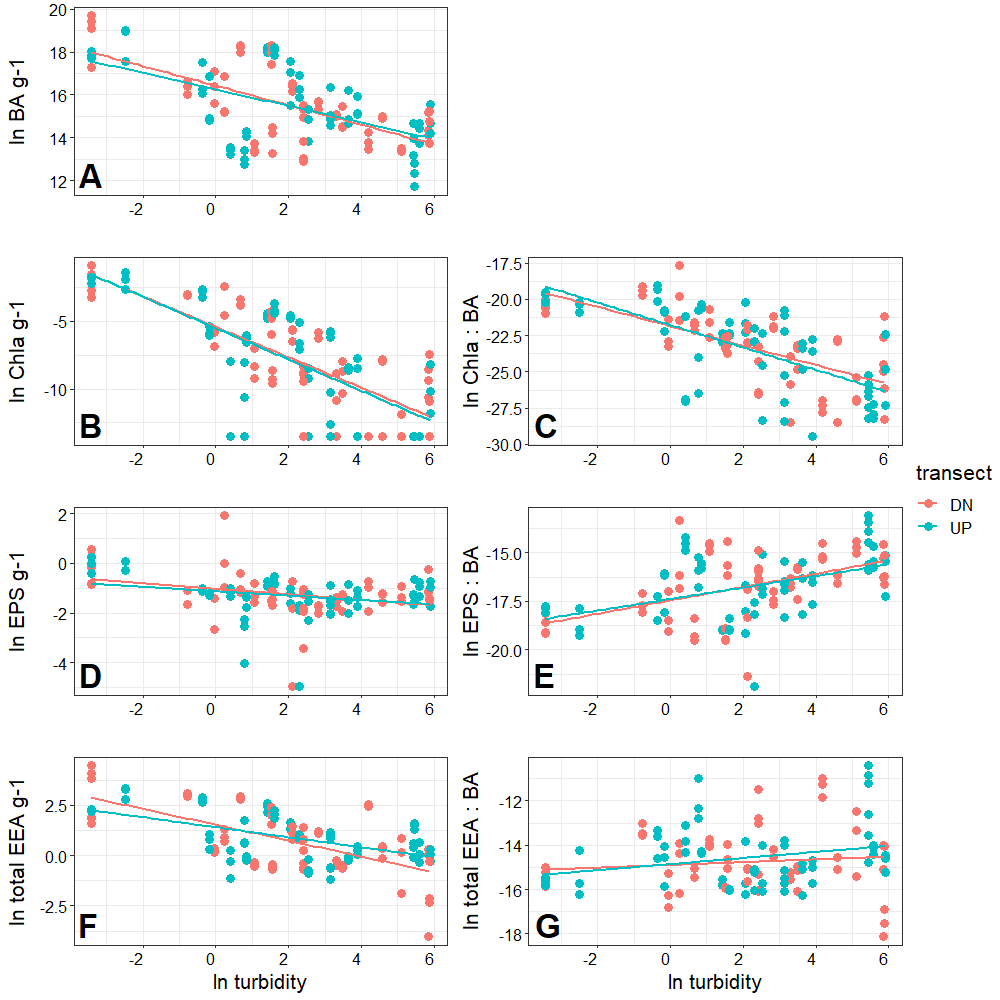

Supplement: Supplementary Table 1 — Characteristics for glacier fed stream biofilms. Reported values are averages and standard deviations calculated from the three patches sampled at each transect. [file Data_Sheet_1.docx]
